# Nature and nurture in fruit fly hearing

**DOI:** 10.3389/fncir.2024.1503438

**Published:** 2024-11-06

**Authors:** Azusa Kamikouchi, Xiaodong Li

**Affiliations:** ^1^Graduate School of Science, Nagoya University, Nagoya, Japan; ^2^Institute of Transformative Bio-Molecules (WPI-ITbM), Nagoya University, Nagoya, Japan; ^3^Department of Molecular, Cellular and Developmental Biology, Neuroscience Research Institute, University of California, Santa Barbara, Santa Barbara, CA, United States

**Keywords:** neural circuit, GABA, dopamine, courtship song, song preference

## Abstract

As for human language learning and birdsong acquisition, fruit flies adjust their auditory perception based on past sound experiences. This phenomenon is known as song preference learning in flies. Recent advancements in omics databases, such as the single-cell transcriptome and brain connectomes, have been integrated into traditional molecular genetics, making the fruit fly an outstanding model for studying the neural basis of “Nature and Nurture” in auditory perception and behaviors. This minireview aims to provide an overview of song preference in flies, including the nature of the phenomenon and its underlying neural mechanisms. Specifically, we focus on the neural circuitry involved in song preference learning, with which auditory experiences shape the song preference of flies. This shaping process depends on an integration hub that processes external sensory stimuli and internal states to enable flexible control of behavior. We also briefly review recent findings on the signals that feed into this integration hub, modulating song preference of flies in an experience-dependent manner.

## 1 Introduction

Humans develop communication skills from early infancy, possessing specialized speech perception abilities long before they produce their first word (Issard et al., 2023). Similarly, many animals have the ability to interpret species-specific communication sounds, which mediate social interactions with important fitness consequences. Acoustic signals are, in fact, widely used in the mate choice of frogs, birds, and insects, playing a crucial role in sexual selection, species recognition, and speciation (Wilkins et al., 2013). Here, the auditory capability to discriminate (and prefer) key sound features is indispensable. This ability is shaped by nature and nurture, involving neural plasticity that tunes the brain to species-specific communication sounds. For example, human newborns show no preference between speech and monkey calls, but by 3 months of age, they begin to prefer speech over monkey calls (Vouloumanos et al., 2010). Human infants further refine their ability to make phonetic distinctions through repeated exposure to their native language. Their brains begin to attune to the native language a few days after birth, influenced by prenatal and/or short-term postnatal exposure (Kuhl et al., 2006; May et al., 2018; Sato et al., 2012). Similarly, juvenile songbirds develop their auditory discrimination abilities during song learning by listening to tutor songs, typically their father’s songs (Yazaki-Sugiyama, 2024). The molecular and neuronal mechanisms underlying this experience-dependent sensory tuning has been studied recently using model animals such as zebra finches (*Taeniopygia guttata*), emphasizing the important role of GABAergic inhibitory circuits (Yanagihara and Yazaki-Sugiyama, 2016).

**Figure 1:**
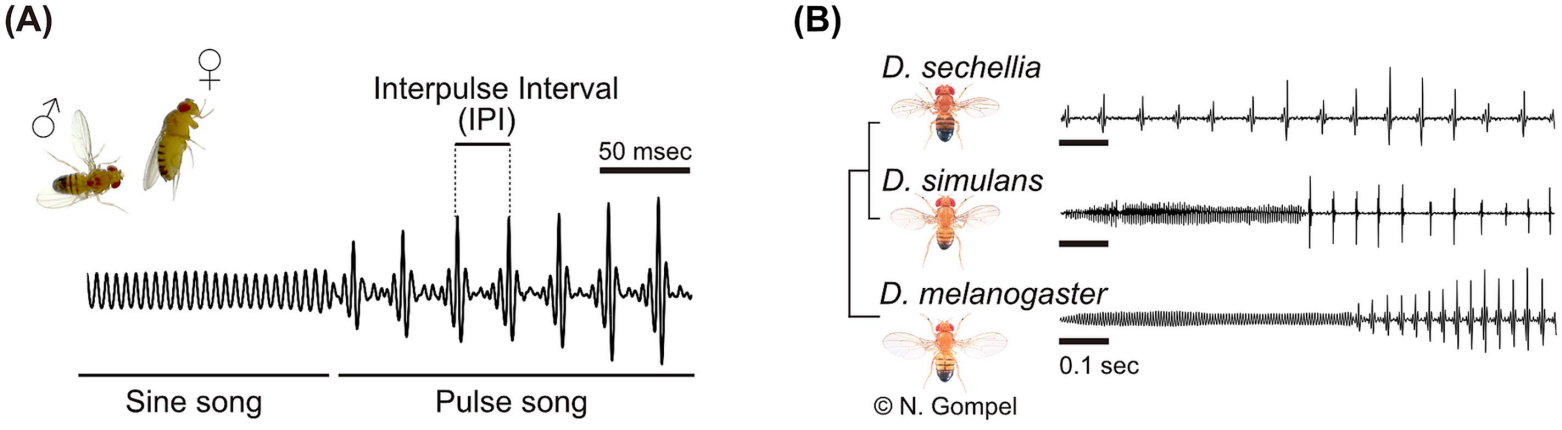
Acoustic communication of fruit flies. (A) The courtship song of *Drosophila melanogaster*. The song consists of two main components: the sine song and the pulse song. The temporal pattern of the pulse song, particularly the interval between pulses, influences the copulation receptivity of female flies. In *Drosophila melanogaster*, the mean interval between two pulses, known as the interpulse interval (IPI), is approximately 35 ms. (B) Different temporal patterns of the pulse song among *Drosophila* species. Fly images and the phylogenetic tree are adapted from Flybase. Fly images were supplied by Dr. Nicolas Gompel. Modified from Kamikouchi and Ishikawa (2016) with permission.

The importance of GABAergic circuits on experience-dependent tuning to a specific communication sound has also been found in the fruit fly *Drosophila melanogaster* (Li et al., 2018). Fruit flies utilize acoustic communication for their mating behaviors, during which the male fly courts a female by vibrating his wings. This generates sound signals so-called the “courtship songs,” typically comprised of several song types (Figure 1A). Among them, the pulse song is the main component that facilitate mating, by increasing the female receptivity for copulation as well as the male’s motivation to chase other flies (Bennet-Clark and Ewing, 1969; von Schilcher, 1976). Importantly, the temporal pattern of the pulse song differs between *Drosophila* species: *D. melanogaster*, *D. simulans*, and *D. sechellia* have the mean intervals between two pulses of 35, 55, and 85 ms, respectively (Figure 1B). Studies using artificial pulse songs have revealed that fruit flies are able to discriminate these inter-pulse intervals (IPIs) (Ohashi et al., 2023; Yoon et al., 2013), shifting the research interest to the following two questions: (1) What the neural mechanism for discriminating IPIs is, and (2) whether the discrimination ability is innate or experience-dependent. In this minireview, we will first overview studies that tackled these questions using the model species, *D. melanogaster*. Then we will focus on plasticity in their song preference, ranging from the phenomenon to the underlying neural mechanisms, including GABAergic inhibitory circuits and dopaminergic signals.

### 1.1 What is the neural mechanism for discriminating IPIs?

The fruit fly (*D. melanogaster*) has a compact brain; less than 1 mm in diameter with approximately 130,000 neurons (Schlegel et al., 2024; Shimada et al., 2005). Recent advancement in automated image acquisition and analysis applied to the ∼100 teravoxel electron microscopy volume of an adult fly brain has yielded comprehensive whole-brain connectome/synaptome datasets of a female fly brain (Dorkenwald et al., 2024). These datasets significantly accelerate investigations on the neuronal mechanisms of sensory processing in the brain.

In flies, sound signals are detected by the antennal ear, which then sends information to the brain via the axons of auditory sensory neurons (i.e., JO neurons) (Kamikouchi et al., 2009). Song information is processed along the auditory pathway, starting from JO neurons to the higher-order neurons in the brain (e.g., pC2 neurons) (Deutsch et al., 2019) that send information to the mating decision center. Along the auditory pathway that processes song information (the song processing pathway hereafter), the neural responses to IPIs are shaped gradually at each step so that the higher-order neurons get more tuned around the species-specific IPI (Wang et al., 2021; Zhou et al., 2015). The first step of this tuning arises between JO neurons and AMMC-B1 neurons, the key secondary auditory neurons in the song processing pathway (Yamada et al., 2018). GABAergic local interneurons are involved in shaping the IPI selectivity of AMMC-B1 neurons, presumably by receiving auditory inputs from JO neurons and in turn providing feedforward inhibition onto AMMC-B1 neurons in the IPI dependent manner.

By utilizing the comprehensive whole-brain connectome/synaptome datasets, the auditory connectome in the fly brain is now established, revealing the entire architecture of the auditory processing circuit (Baker et al., 2022). This serves as the basis to fully explore the neural substrates that shape the gradual IPI tuning along the song processing pathway. In frogs and crickets, excitatory and inhibitory inputs are suggested to interact to generate IPI selectivity (Hedwig, 2016; Rose, 2014). Such interactions are frequently observed in the auditory connectome of the fly brain (Baker et al., 2022), suggesting a shared neuronal mechanism to generate IPI selectivity across animal phyla.

### 1.2 Is the discrimination ability innate or experience-dependent?

Although males are the singer and the females are the listener (and evaluator) during the mating ritual, both male and female flies show behavioral responses when they detect the song: Male flies increase their locomotor activity and chase other flies, possibly due to male–male competition, while female flies slow down their locomotion (Bennet-Clark and Ewing, 1969; von Schilcher, 1976). These behavioral changes occur only by pulse songs carrying a specific IPI range, which matches to the statistical characteristics of male songs (Deutsch et al., 2019; Ohashi et al., 2023; Yoon et al., 2013). Interestingly, group housing of males sharpens their IPI preference when compared to the single-housed flies (Li et al., 2018), which aligns with studies indicating that social experience affects courtship behavior in *Drosophila* (Ellis and Kessler, 1975; von Schilcher, 1976). Furthermore, broadcasting the song via a loudspeaker to individually housed flies (a procedure referred to as “training”) is sufficient to tune their IPI preference (Li et al., 2018). During this training, flies are isolated in individual chambers and exposed to an artificial pulse song with a species-specific IPI of 35 ms. After 6 days of training, their song preferences were tested, revealing that the trained flies had become tuned in their song-response behavior by significantly reducing their behavioral response to a song with a heterospecific IPI of 75 ms. This contrasts with the behavioral phenotype of naïve flies who never experienced the training. Because fruit flies gather in groups at feeding sites (Soto-Yéber et al., 2018), group-housed males are likely exposed to the song of other conspecific males. A field study revealed that male–female courtship, and occasionally male–male courtship, occurs frequently (Dukas, 2020), which might promote preference tuning in the wild. This hypothesis is supported by a subsequent finding that having intact wings are necessary for group-housing males to develop their IPI preference (Li et al., 2018).

In vertebrate species with vocal communication, early auditory learning is more effective when acoustic training is accompanied by social interactions with a live adult tutor (Katic et al., 2022). In contrast, in flies, social interactions are not necessary to tune their IPI preference (Li et al., 2018). This experience-dependent tuning of the song response behavior occurs both in male (by group housing or via loudspeaker) and female flies (via loudspeaker) and is referred to as “song preference learning” in flies. Although it is unclear whether this experience-dependent behavioral change is due to increased discrimination ability or preference refinement, this finding highlights the remarkable plasticity of the fly’s auditory system to process acoustic communication signals.

It’s noteworthy that prior exposure to songs with a heterospecific 75-ms IPI does not affect song preference of flies, indicating the existence of a specific “IPI window” to establish song preference learning (Li et al., 2018). In the wild, *Drosophila* meet and mate on fermenting fruits where a diversity of species and sexes congregate (Dukas, 2020; Soto-Yéber et al., 2018). To avoid untuning their song preference, fruit flies presumably have the innate ability to discriminate between songs with different IPIs, and only the experience of hearing conspecific songs can tune their behavioral response to these variations.

### 1.3 Neural circuit mechanisms underlying song preference learning in fruit flies

In vertebrates, the maturation of the excitation-inhibition balance that governs sound perception requires acoustic experience, during which the GABAergic system plays a significant role. In mammals, auditory experience mediates the maturation of GABAergic inhibition, which fine-tunes sound perception in the auditory cortex, while hearing loss hinders this process (Dorrn et al., 2010; Kotak et al., 2008). In songbirds, experience-dependent recruitment of GABA-mediated inhibition shapes auditory cortical circuits (Yanagihara and Yazaki-Sugiyama, 2016). Consistent with these vertebrate systems, studies in female flies have also identified GABA as a crucial component of song preference learning.

**Figure 2:**
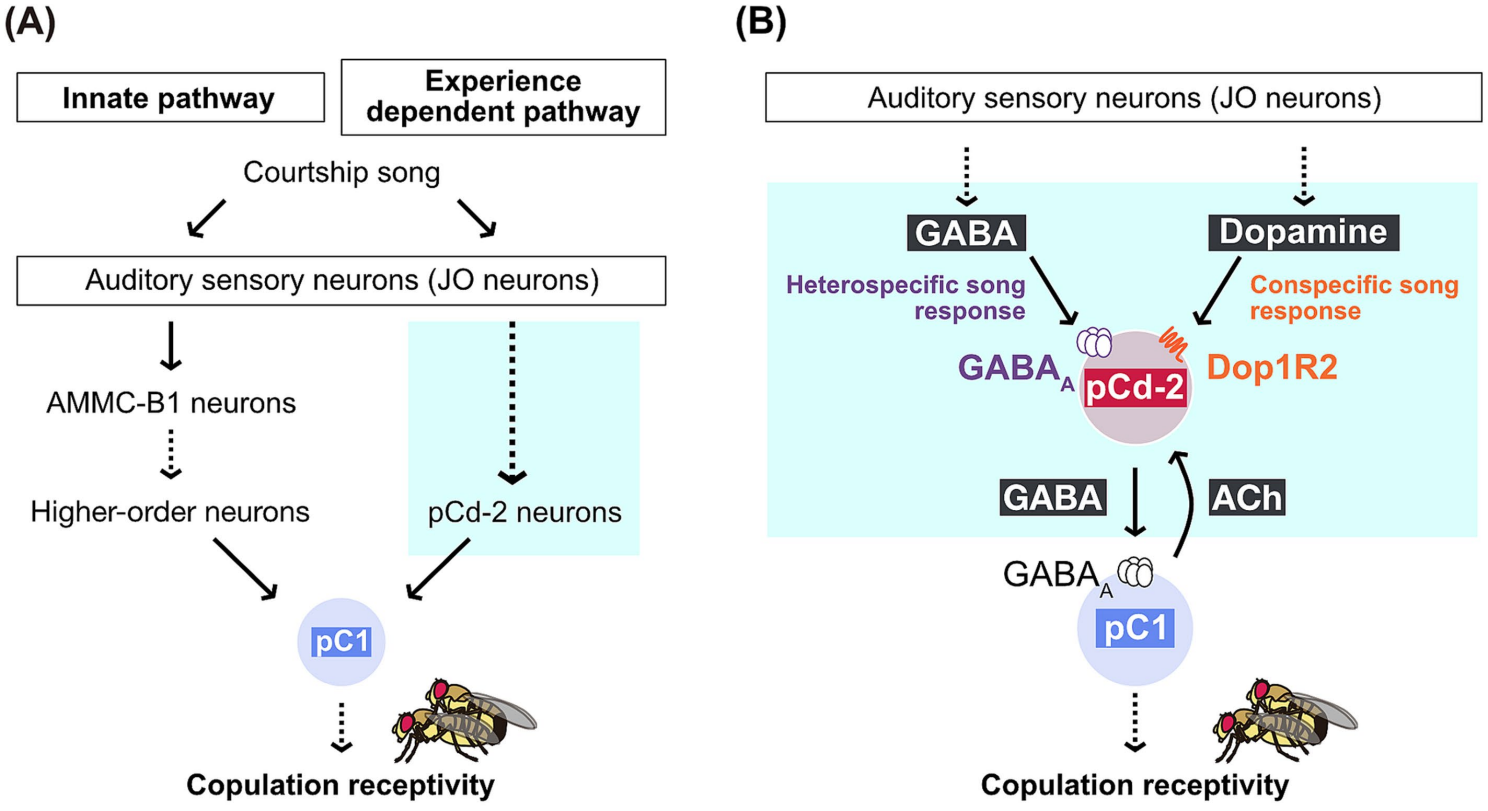
Neural circuit model for song preference learning. **(A)** Female song responses are regulated by the interaction between innate and experience-dependent pathways, both of which originate from auditory sensory neurons and converge at pC1 neurons in the brain. pC1 neurons play a crucial role in regulating female mating behavior by increasing copulation receptivity when activated. The detailed circuit mechanism in the blue box is shown in panel B. **(B)** A model for the neural circuit mechanism of experience-dependent modulation. Song preference learning in flies involves two mechanisms: Suppressing the response to heterospecific songs and maintaining the response to conspecific songs after experience. These mechanisms are likely mediated by GABA and dopamine, which are transmitted to pCd-2 neurons respectively. pC1 neurons are excitatory cholinergic neurons that transmit acetylcholine. Modified from Imoto et al. (2024) with permission.

In female flies, pC1 neurons in the brain regulate copulation receptivity by integrating courtship-related sensory stimuli, such as pheromones and courtship songs (Zhou et al., 2014). Besides being a hub to regulate copulation acceptance, female pC1 neurons play a key role in song preference learning: Disruption of GABAergic signals to pC1 neurons abolishes song preference learning, failing to suppress the response to the heterospecific 75-ms IPI song after acoustic experience of hearing conspecific songs (Li et al., 2018). These GABAergic signals are presumably transmitted by pCd-2 neurons, one cluster of GABAergic neurons that form reciprocal synaptic connections with pC1 neurons in the brain (Imoto et al., 2024). Interestingly, pC1 neurons and pCd-2 neurons both express the sex-specific transcription factor *doublesex* (*dsx*), which plays a role in somatic sexual differentiation in insects. The reciprocal circuits between these two types of *dsx-*expressing neurons in the brain (i.e., pC1 and pCd-2 neurons) are proposed to function as a hub integrating sensory signals and internal states, enabling flexible control over female copulation. Thanks to the EM connectome database, the neuronal circuit consisting of pCd-2 neurons (four per hemibrain) and pC1 neurons (five per hemibrain) has been comprehensively identified at synaptic resolution, allowing researchers to propose a neural circuit model for song preference learning. In this model, female song responses are regulated by the interaction between innate and experience-dependent pathways, both originating from auditory sensory neurons (Figure 2A) (Li et al., 2018). The innate pathway relays song information to pC1 neurons, ultimately regulating female receptivity, while GABAergic pCd-2 neurons in the experience-dependent pathway interact reciprocally with pC1 neurons, gating the song response based on prior sound experiences (Imoto et al., 2024). Using a combination of single-cell transcriptome data (Davie et al., 2018) and molecular genetics, it has been further suggested that pCd-2 neurons receive GABAergic and dopaminergic signals to regulate experience-dependent song responses (Figure 2B). GABAergic signals to pCd-2 neurons are likely necessary to suppress the behavioral response to heterospecific songs, while dopaminergic signals help maintain the response to conspecific songs after prior exposure to conspecific songs (Imoto et al., 2024).

pC2 neurons are another cluster of *dsx-*expressing neurons in the *Drosophila* brain, which are tuned for multiple temporal aspects of the pulse song and drive sex-specific behaviors in both males and females. Notably, group housing of male flies sharpens IPI preference of pC2 neurons (Deutsch et al., 2019), a similar phenomenon observed in song preference learning. pC2l, the lateral cluster of pC2 neurons, has synaptic outputs to some pC1 neurons (pC1d/e) in females (Baker et al., 2022), suggesting an involvement of pC2 neurons in regulating the activity landscape of the pC1/ pCd-2 hub system.

## 2 Discussion

A broad range of sensory skills improve with practice and training. Particularly during development, including pre- and postnatal stages, the effect of experiences to improve discrimination ability is especially strong, as exemplified in language acquisition of infants during a specific period referred to as the sensitive period. Song preference learning in flies shares many common aspects with such ability improvement in humans and other vertebrates, including the interaction of nature and nurture and re-balance of inhibition and excitation. At the molecular level, both gerbils and fruit flies show involvement of GABA_A_ receptors in enhancing sound discrimination abilities through training (Caras and Sanes, 2017; Imoto et al., 2024; Li et al., 2018). Additionally, the interaction between dopaminergic and GABAergic signals modulates auditory plasticity in both songbirds and fruit flies, illustrating a similarity at the circuit level (Macedo-Lima et al., 2021). Expanding the recently proposed fly model of song preference learning will provide a simple and manipulatable model for studying the mechanisms underlying auditory learning in general.

However, several open questions remain in the fly model: Where do the GABAergic and dopaminergic signals to pCd2 neurons originate? By what mechanism does the acoustic experience of hearing conspecific songs recruit these neurotransmitter signals? Are specific synaptic pathways in the pC1/ pCd-2 reciprocal circuit potentiated or inhibited following acoustic experiences to produce different behavioral outcomes? How do neurons in the song processing pathway, such as pC2 neurons, interact with the pC1/ pCd-2 reciprocal circuit? Answering these questions will help create a detailed map of structural and functional connections underlying song preference learning, thereby improving the model’s utility. Moreover, further explorations of the transcriptome in key neurons during this dynamic process might help identify new or unique molecules, which could then serve as candidate targets for manipulating learning in flies and, hopefully, in other animals and humans.
